# A transfer learning framework based on motor imagery rehabilitation for stroke

**DOI:** 10.1038/s41598-021-99114-1

**Published:** 2021-10-05

**Authors:** Fangzhou Xu, Yunjing Miao, Yanan Sun, Dongju Guo, Jiali Xu, Yuandong Wang, Jincheng Li, Han Li, Gege Dong, Fenqi Rong, Jiancai Leng, Yang Zhang

**Affiliations:** 1 School of Electronic and Information Engineering (Department of Physics), Qilu University of Technology (Shandong Academy of Sciences), Jinan, 250353 China; 2 School of Electrical Engineering and Automation, Qilu University of Technology (Shandong Academy of Sciences), Jinan, 250353 China; 3 Department of Physical Medicine and Rehabilitation, Qilu Hospital, Cheeloo College of Medicine, Shandong University, Jinan, 250012 China; 4 Shandong Energy Group Co Ltd., Jinan, 250014 China

**Keywords:** Biomedical engineering, Neuroscience

## Abstract

Deep learning networks have been successfully applied to transfer functions so that the models can be adapted from the source domain to different target domains. This study uses multiple convolutional neural networks to decode the electroencephalogram (EEG) of stroke patients to design effective motor imagery (MI) brain-computer interface (BCI) system. This study has introduced ‘fine-tune’ to transfer model parameters and reduced training time. The performance of the proposed framework is evaluated by the abilities of the models for two-class MI recognition. The results show that the best framework is the combination of the EEGNet and ‘fine-tune’ transferred model. The average classification accuracy of the proposed model for 11 subjects is 66.36%, and the algorithm complexity is much lower than other models.These good performance indicate that the EEGNet model has great potential for MI stroke rehabilitation based on BCI system. It also successfully demonstrated the efficiency of transfer learning for improving the performance of EEG-based stroke rehabilitation for the BCI system.

## Introduction

The brain-computer interface (BCI) is a communication system that can directly measure brain activities related to users' intentions and convert them into control signals^1^. In recent years, the BCI system has been widely used in the medical field, such as in the rehabilitation of stroke patients^2^. Among different methods of brain activity monitoring, electroencephalography (EEG) technology provides a simple and non-invasive solution for the BCI system and has been used in many BCI studies^3^. Generally speaking, a BCI system can generally be divided into four modules: signal acquisition, signal processing, control equipment, and feedback^4^. The three most common BCI paradigms based on EEG are as follows: P300 evoked potentia^5^, steady-state visual evoked potentials (SSVEP)^6^ and motor imagery (MI). In the absence of muscle contraction, the MI procedure includes changes in the movement rhythm activated by the cerebral cortex^7^. In this paper, data comes from stroke patients with motor dysfunction. Rehabilitation therapy based on the MI BCI system can repeatedly stimulate the damaged motor cortex to reactivate the motor nerve cells around the damaged cells and partially restore the patients’ motor function.

In the field of biomedical engineering, the application of deep learning algorithms has become more and more extensive in many technologies^8^. In terms of BCI, the EEG signal feature extraction, classification, and recognition methods have received extensive attention^9^. Different types of layers can be built into different neural networks. The common layers include fully connected layers, convolutional layers, and hidden layers. These layers can be constructed as fully connected networks^10^, Convolutional Neural Networks (CNN)^11^ or Recurrent Neural Networks (RNN)^12^. Deep learning is an algorithm for the high-level abstraction of data using multiple processing layers consisting of complex structures or multiple nonlinear transformations. Deep learning is a machine learning method that allows the computer to keep trying until it finally gets close to the task object. Traditional machine learning technologies, such as Support Vector Machine (SVM), Linear Discriminant Analysis (LDA), and Common spatial pattern (CSP) algorithms have achieved good results. Siuly and Li have designed a least squares SVM method to classify two types of MI signals^13^. Ashok et al. have proposed two weighted CSP task classification methods, and achieved more accurate classification^14^. In recent years, EEG classification based on deep networks can beat traditional methods on large datasets. Compared with traditional classification methods, deep learning methods can describe nonlinear features without manual assistance. This makes the deep learning method an important choice for processing MI signals based on BCI. Some recent studies have used different deep learning techniques to automatically extract features from EEG data. Tabar and Halici have proposed a CNN with a stacked autoencoder (SAE) that can achieve better classification accuracy than traditional classification methods on the BCI competition IV-2b data set^15^. Lu et al. have proposed a deep belief network classification method using restricted Boltzmann machines (RBM)^16^. Sakhavi et al. have introduced the envelope representation of EEG by using Hilbert transform, and developed a new MI-based BCI classification framework through CNN. They have applied the algorithm to the BCI competition IV-2 data set, and beat the most advanced classification accuracy reported so far^17^. Robinson et al. have used a deep CNN representation of multi-band, multi-channel EEG input to further improve the accuracy^18^. Zhao et al. have developed a new 3D representation of EEG, a multi-branch 3D CNN and corresponding classification strategy. Their method has achieved good performance^19^. In theory, deep learning can achieve more effective EEG feature extraction and higher precision pattern classification^20^. However, due to the poor physical condition and high prevalence of stroke patients, the signal acquisition can be difficult, which has an impact on the construction of large-scale datasets. The use of deep learning algorithms for MI research in stroke patients is limited. In this paper, our algorithm employs the deep transfer learning method, which can effectively solve the above problems. The purpose of transfer learning is to apply knowledge or patterns learned from one task to other different but related tasks^21^. Transfer learning is achieved by passing constant or exchanging differentiated information between subjects. Features extracted by transfer learning have similarities and inheritance^22^. These characteristics can be specified not only in a certain dataset but also in other related datasets. That can ensure the effectiveness of EEG's deep network transfer learning^23^.

In this work, the study introduces multiple deep learning neural network models for transfer learning. To improve the performance of the BCI system for the rehabilitation of stroke patients, this study applies these neural networks to analyze the EEG of stroke patients. The proposed algorithm is to combine EEGNet^24^ or other neural network models with ‘fine-tune’^25^ to identify MI tasks. This study adopts the method of learning within the subject to evaluate the performance of all frameworks. By comparing the experimental results of all models, it can be inferred that EEGNet is the best network model for transferring learning in all frameworks. The average accuracy of this model reaches 66.36%. During the experiment, ‘fine-tune’ can save time in the training process and reduce the complexity of the algorithm. The experiment shows that transfer learning can effectively improve the performance of the BCI system for the rehabilitation of stroke patients, and also proves that the proposed framework is effective and robust.

The remaining of this article are as follows: Section II Methods introduces the experimental dataset and different deep learning models for MI-based BCI system. Section III Results describes the classification performance, methods comparison and complexity of the proposed algorithm. Finally, Section IV Discussion summarizes the conclusion.

## Methods

### Experimental data

**Figure 1:**
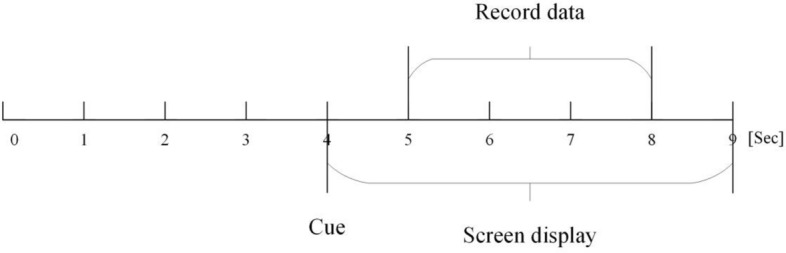
The collection process of an experiment.

The EEG dataset, which comes from the Department of Physical Medicine & Rehabilitation, Qilu hospital, Cheeloo College of medicine, Shandong University, is taken from 11 subjects (6 healthy people and 5 patients). The experiment uses a 64-channel NeuroScan EEG acquisition equipment to collect data from stroke patients (EEG data including MI). A complete experimental process takes about 9 seconds, the time of one trial is shown in Figure 1. The experiment starts with 4 seconds of the resting-state EEG. Following a cue, EEG signal including MI tasks have been recorded. There are cues at the beginning and end of MI, respectively. The sampling frequency of signal acquisition is 1000 Hz. During the collection process, subjects follow the on-screen prompts to perform imaginary movements, including left-hand and right-hand grasping. The time displayed by the cue on the screen is 5 seconds. The imagined time for each trial is 3 seconds, and it only contains one type of action. The interval between the two trials is 4 seconds. During the whole experiment procedure, the visual cues of the left and right hands are random. The whole experiment consisted of 30 left-hand motor imaginations and 30 left-hand motor imaginations.

After receiving a detailed explanation of the purpose and potential risks of the experiment, all participants provided written informed consent. The study protocols have been approved by the medical ethics committee of Qilu Hospital, Cheeloo College of Medicine, Shandong University. The study has carried out in accordance with relevant guidelines and regulations.

### Data preprocessing

Preprocessing includes filtering and downsampling. In this study, an 8–30 Hz Butterworth band-pass filter is used to eliminate noise^27^, and then the data sampling frequency is reduced from 1000 to 100 Hz.

The EEG dataset is stored in 3D format (M, C, T), where M is the number of trials. This study uses the stroke patients’ EEG dataset that includes two types of MI tasks (including left-hand and right-hand tasks). Dividing the data of each subject into a training set and a test set. The data of each subject is classified into the training set and test set. Each subject has collected a total of 60 trial data, and each trial data represents a MI task. For each subject, 40 trial data are used as the training set, and the remaining 20 trial data are used as the test set. Randomly assigned, using 10 cross-validation to get the average accuracy of each person. A01, A02, A03, A05, A07, A11 are healthy people. A04, A06, A08, A09, A10 are patients.

### Deep learning models

#### EEGNet architecture

**Figure 2:**
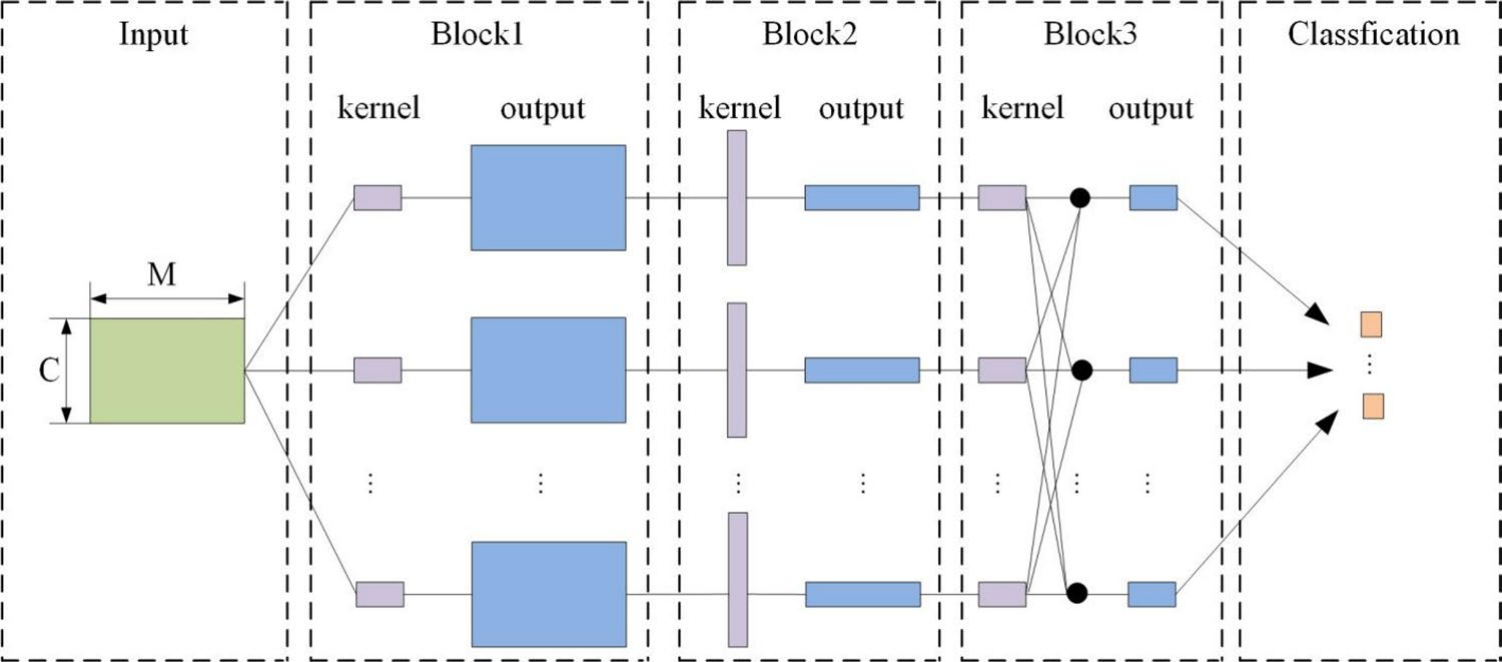
The overall visualization of the EEGNet structure. The line represents the connectivity of the convolution kernel between input and output (called feature map). Where, C is the number of channels, T is the number of sampling points.

EEGNet is a compact CNN architecture for processing EEG. It can be trained with very limited data, and it can produce a neurophysiological explanatory function. Figure 2 and Table 1 respectively describe the visual structure and specific parameters of the EEGNet model. The input layer size of the model is (C, T), C represents the number of channels, T represents the number of sampling points for each channel. This study uses Adam optimizer^28^ and minimizes the categorical cross-entropy loss function.

**Table 1:** Parameter setting of EEGNet structure, $F_{1}$ = number of temporal filters, $D$ = depth multiplier, $F_{2}$ = number of pointwise filters.

Block	Layer	Filters	Size	Output	Activation
1	Input			$C \times T$	
	Reshape			$1 \times C \times T$	
	Conv2D	$F_{1}$	(1, 64)	$F_{1} \times C \times T$	Linear
	BatchNorm			$F_{1} \times C \times T$	
	DepthwiseConv2D	$D \times F_{1}$	$(C,{\kern 1pt} {\kern 1pt} {\kern 1pt} {\kern 1pt} {\kern 1pt} 1)$	$(D \times F_{1} ) \times 1 \times T$	Linear
	BatchNorm			$(D \times F_{1} ) \times 1 \times T$	
	Activation			$(D \times F_{1} ) \times 1 \times T$	ELU
	AveragePool2D		(1,4)	$(D \times F_{1} ) \times 1 \times T/4$	
	Dropout		p = 0.25 or p = 0.5	$(D \times F_{1} ) \times 1 \times T/4$	
2	SeparableConv2D	$F_{2}$	(1,16)	$F_{2} \times 1 \times T/4$	Linear
	BatchNorm			$F_{2} \times 1 \times T/4$	
	Activation			$F_{2} \times 1 \times T/4$	ELU
	AveragePool2D		(1,8)	$F_{2} \times 1 \times (T/32)$	
	Dropout		p = 0.25 or p = 0.5	$F_{2} \times 1 \times (T/32)$	
	Flatten			$F_{2} \times T/32$	
Classifier	Dense	$N \times (F_{2} \times T//32)$	max norm = 0.25	$N$	Softmax

In Block 1, starting from the input layer, the module includes two convolution steps. Firstly, using a 2D convolution and the filter to output feature map (the feature map contains EEG signals with different frequencies), then perform batch normalization. Secondly, using deep 2D convolution to learn about spatial filters, then perform batch normalization. In the field of pattern recognition, deep convolution has the advantage of reducing the number of trainable parameters to be fitted. Because it does not need to connect all previous feature maps. Using the combination of Conv2D and Depthwise Conv2D, spatial filters of specific frequencies can be effectively extracted. $D$ is responsible for controlling the number of spatial filters for each feature map. Dropout is used to prevent overfitting^29^. An average pooling layer is used to reduce the number of features.

In Block 2, a separable convolution method is used. The first is a deep convolution with a kernel size of (1, 16), then using a separable convolutional layer. There are two advantages to using separable convolution: 1) Reduce the number of parameters to be fitted. 2) Learn the feature kernel to separate the relationship with the feature map, and summarize each feature map to obtain the best combination output. The average pooling layer is used to reduce the number of features.

In the classification block, the features extracted from the first few layers are passed to the softmax classification layer with N units (N is the number of MI tasks)^30^.

#### Other structures

- *DenseNet model *DenseNet cannot perform convolution and pooling operations on each layer, instead, using dense block as a unit selectively. There is only one convolution layer before the first dense block. In other dense blocks, they are first convolved and then pooled, which makes DenseNet has good performance^31^.
- *Xception model *The Xception model is further optimization of the Inception^32^ model. Many neural networks divide the input data into several compressed data blocks for convolution. However, Xception is different from other neural network models. To obtain channel correlation, it maps spatial correlation for each output channel separately, then performs convolution with a depth of 1 × 1^33^.
- *ResNet50 model *ResNet has 2 basic modules: one is Identity Block, the input and output dimensions of this module are the same, therefore, multiple ones can be connected in series. The other is Conv Block, the input and output dimensions of this module are different, so they cannot be connected in series. The role of Conv Block is to change the dimension of the feature vector, in other words, it transforms the input into a small but deep feature map. Conv Block usually uses a unified and relatively small core. As the depth of the ResNet network increases, the learned features are getting more and more complex. Therefore, before entering the Identity Block, Conv Block needs to be used to convert the output sizes to continuously connect to Identity Block^34^.
- *VGG16 model *In the VGG16 model, three 3 × 3 convolution kernels are used to replace the 7 × 7 convolution kernels of the AlexNet network^35^, and two 3 × 3 convolution kernels are used to replace the 5 × 5 convolution kernel of the AlexNet network. The purpose is to ensure that they have the same perception field^36^. Enhancing the depth of the neural network can improve the performance of different types of neural networks.

First of all, the difference between EEGNet and other models is that EEGNet can not only realize time/space convolution but also perform frequency domain analysis. Other models can only implement time/space convolution. Secondly, EEGNet uses a separable convolution layer, which saves computation.

### Combination of ‘fine-tune’ and EEGNet

The effectiveness of transfer learning depends on many factors. Among them, the most important factor is the similarity between the original data and the target data. The higher the similarity, the better the ‘fine-tune’ effect. The features obtained by the first few layers of EEGNet are the basic general features (for example, extracting a specific frequency spatial filter from the first few layers). The latter layers extract specific features (for example, the model can summarize the kernel of each feature map separately and find the best combination of feature maps). In this experiment, the size of the dataset is relatively small. To avoid over-fitting, the ‘fine-tune’ of the proposed neural network is divided into the following steps:

- Modify the output parameters of the last layer. The proposed method is to freeze or retrain the parameters of the first few layers, and then to modify the category parameters of the softmax layer.
- Adjust the configuration parameters of the model to appropriately reduce the learning rate^37^, step size, and epoch. The learning rate of the model is relatively low because the effective model weights are used for ‘fine-tune’. If the learning rate is too high, the model can update quickly and destroy the original good weight information. After ‘fine-tune’, this study chooses to open all layers and update the step size parameters. The EEGNet model was previously performed on the large-scale dataset, which invisibly expanded the trained EEG data, and its processing performance is very beneficial to the dataset. Therefore, ‘fine-tune’ can improve the model to obtain better results after relatively few epochs.
- Start training and load the parameters of the pre-trained model.

The proposed framework not only uses the EEG feature extraction principle encapsulated in the pre-training model but also employs ‘fine-tune’. This makes the model more robust and generalized. The process of adapting the parameters aims to obtain an EEG signal analysis model suitable for MI recognition.

The two ‘fine-tune’ methods have been employed. The first method is the weights of the pre-trained model are randomly initialized. New training datasets are fed in the neural network for retraining. The second method is some weights are used on the previous layers, the weights on the back layers are initialized. Finally, according to the experimental results, the optimal combination of the frozen layers and the retrained layers has been found.

## Results

### Classification performance

**Figure 3:**
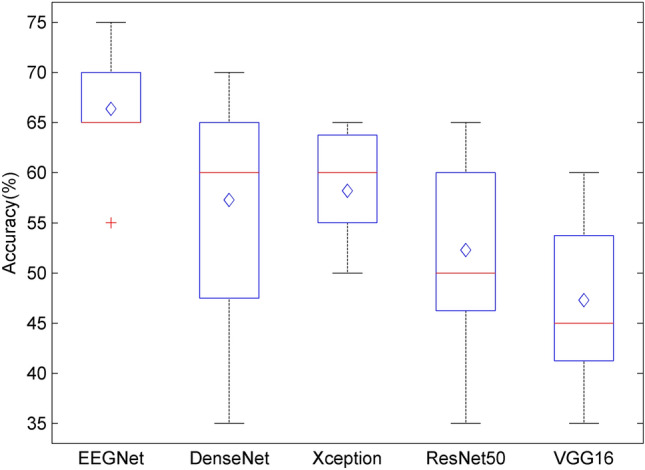
The overall average classification accuracy of all models.

**Figure 4:**
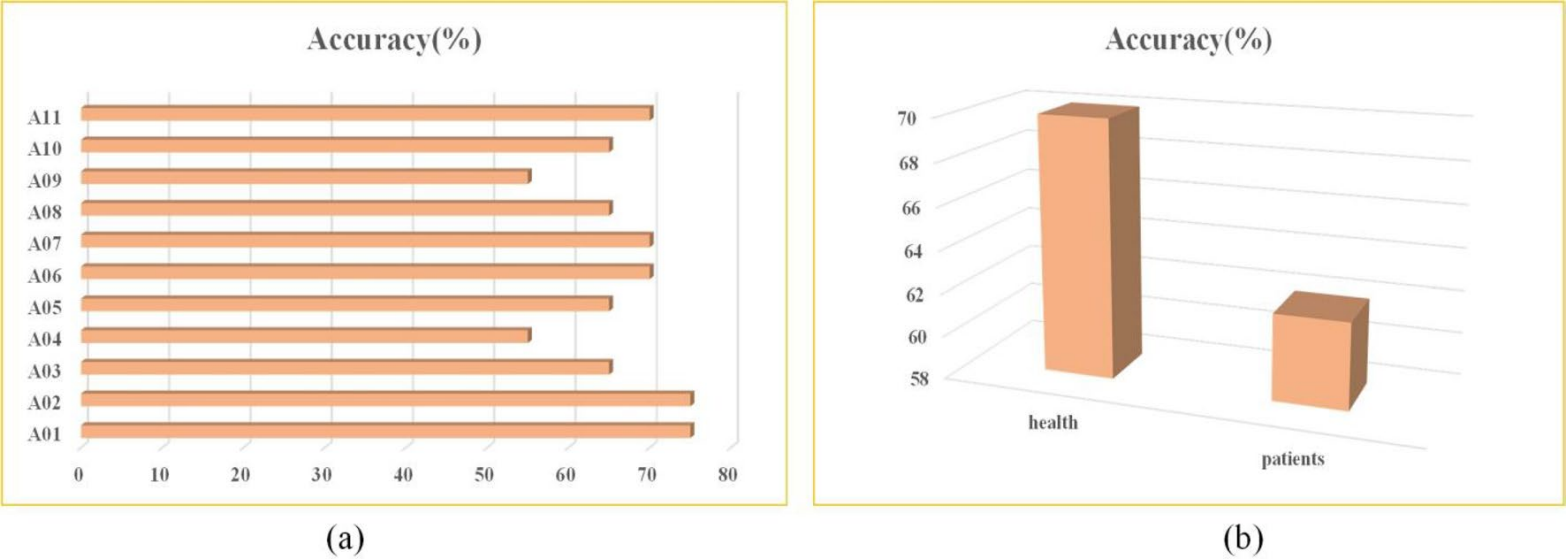
(**a**) The highest accuracy of EEGNet for each subject. (**b**)Average accuracy of the two datasets (health and patients).

This study feeds the training dataset and test dataset of each subject into all neural network models. Figure 3 is the display of the overall average accuracy of each model. As can be seen from the figure that the EEGNet model can get the best performance. The average classification accuracy of the EEGNet model among subjects is 66.36%. At this time, the parameters of the model are shown in Table 2. As shown in Fig. 4, we show the overall average accuracy of healthy people and patients. We have tested the statistical significance of the classification accuracies corresponding to the 11 subjects of the SVM、LDA, and our proposed framework, and got p of $7.13 \times 10^{{{ - }5}}$. It can be seen that the p-value is less than 0.05, so the classification accuracies have significant differences. It proves that the improvement of our proposed method is statistically significant. For the experimental EEG data, the classification performance of SVM, LDA classifier, and our proposed framework have been compared, and the results are shown in Table 3. It can be seen from the performance of our proposed framework is better and more effective than traditional classifiers.

**Table 2:** The value of each parameter of the model.

Parameters	Value
Learning rate	0.0001
Dropout	0.5
Epoch	100
$F_{1}$	4
$F_{2}$	8
$D$	2

**Table 3:** Classification results obtained by different ‘fine-tune’ methods.

Subject	Proposed (%)framework	SVM (%)	LDA (%)
A01	75	65	60
A02	75	75	70
A03	65	55	65
A04	55	60	45
A05	65	50	60
A06	70	55	65
A07	70	60	65
A08	65	55	55
A09	55	60	50
A10	65	65	65
A11	70	65	50
Mean	66.36	60.45	59.09

### Methods comparison

Three processing methods have been performed on the EEGNet model. The first method is to randomly initialize the weights of the whole network, then a new training dataset is put in for retraining. (the processed model is called EEGNet_0).

The second method is to freeze the weights of Block 1 in the pre-trained model and retrain the rest of the following layers so that new weights can be obtained (the processed model is called EEGNet_1).

The third method is similar to the second method, except that the layer weights of Block 1 and Block 2 are frozen, and the rest is the same (the processed model is called EEGNet_2).

The three pre-training models have been compared. The average classification accuracies of all subjects have been described in Table 4. It shows the results of the different ‘fine-tune’ methods.

**Table 4:** Classification results obtained by different ‘fine-tune’ methods.

Subject	EEGNet_0 (%)	EEGNet_1 (%)	EEGNet_2 (%)
A01	65	75	70
A02	60	75	65
A03	55	65	60
A04	55	55	55
A05	60	65	60
A06	65	70	65
A07	65	70	65
A08	60	65	60
A09	55	55	60
A10	55	65	60
A11	55	70	65
Mean	59.09	66.36	62.27

It can be seen from Table 4 that the classification results of the EEGNet_1 model are higher than those of the other two. The experimental results show that the method of partially freezing the weights is better than the method of the whole neural network initialization. It can be seen from Table 4 that the classification result of the EEGNet_1 model is better than that of the EEGNet_2 model. Because the extracted features in Block 2 are the specific features associated with MI. By freezing the weights of the Block 2 layer, the ability of network training is reduced, so the performance is not good.

Finally, the optimal results obtained from the second model are as follows: 1) In the EEGNet model, it can be seen that the general features can be extracted in Block 1, and Block 2 can extract the specific features. 2) Compared with initializing the weights of the entire network, the classification result of transfer learning is better.

### Algorithm complexity

In deep learning, computational complexity is one of the criteria for measuring algorithm performance. The innovations of many models are developed around the optimization of complexity, and the basic principle is to turn multiplication into addition. In this paper, the study calculated the FLOPs and Bytes of each model separately to measure the time complexity and space complexity of the proposed algorithm^26^.

FLOPs represent the number of floating-point operations and determine the training/prediction time of the model. If the complexity is too high, it can cause model training/prediction to consume a lot of time, and it is impossible to quickly verify ideas and improve the model, nor can it achieve rapid prediction.

Bytes focuses on measuring the independence of hardware functional modules in the process of implementing algorithms. Bytes measure the number of parameters of the model. Due to dimensional limitations, the more parameters of the model, the larger the amount of data required to train the model. The dataset in real life is usually not too large, which makes the training of the model easier to overfit.

In this paper, FLOPs and Bytes of all models are used to analyze the algorithm complexity, as shown in Table 5. It can be seen from the table that the time complexity and space complexity of EEGNet are far lower than other models, thereby reducing the number of operations and the number of parameters. The training time of the EEGNet model is also much less than other models.

**Table 5:** FLOPs, Params and times of all models.

Model Type	FLOPs(G)	Params(M)	Times(s)
EEGNet	0.0041	0.013	176
DenseNet	2.87	25.56	440
Xception	5.73	7.98	396
ResNet50	4.11	23.83	506
VGG16	18.11	138.36	792

## Discussion

In this paper, the purpose of the research is to determine whether the combination of the EEGNet model and ‘fine-tune’ can be effectively used for limited EEG data size. The proposed framework is mainly employed for the EEG dataset of healthy people and patients. The results of the experiment can be seen that the overall classification results of healthy people are better than those of patients. It is even more challenging to acquire EEG from stroke patients. Collecting EEG data from stroke patients is a difficult and costly process, because they may have trouble sitting still and avoiding blinking or head/body movements that often contaminate the recorded EEG. Furthermore, brain injury will seriously change the dynamic characteristics of EEG signals, thus increasing the instability of data distribution. It is a hot topic to obtain a large quantity and high-quality EEG data from patients. Finally, the performance of patients' brain activity may not achieve the expected effect. which are potential factors affecting the final results.

This study analyzes the EEG signal of all subjects to explain the effectiveness of information transmission of transfer learning. These neural network models are inspired by computer vision and learn to extract effective features. The general features of all samples can be trained through the first few layers of the model. At a deeper level, learn more specific features related to experimental tasks. This work can train smaller datasets by freezing the previous layers because transfer learning can reduce the number of network parameters that must be optimized. This study proves that the proposed model can transfer certain knowledge for the same paradigm. The performance indicates that the sharing of neural network models should be encouraged in the field of EEG analysis. Sharing the neural network can further enable the model to train with more data. By freezing or retraining a specific number of layers, the neural network can be reused for different MI tasks. Ultimately, this study can improve the overall performance of the model and expand its application in the BCI field. Besides, it can be found from the experimental results that the EEGNet performs better than other neural network models in MI recognition. The experimental results show that although only a small amount of dataset is trained, the knowledge of features has been effectively learned from the EEG data of stroke patients through a transfer learning strategy.

The pre-trained EEGNet is a model obtained through repeated training of a large amount of EEG datasets, which makes the model more robust. EEGNet combined with transfer learning can be used to reduce computational complexity. Experiments prove that ‘fine-tune’ can be utilized to improve the performance of the proposed architecture. It can be inferred that the proposed framework can transfer relevant knowledge to identify different MI tasks. In future work, a large amount of EEG signals will be used to monitor EEG-biomarkers and evaluate system performance. At the same time, we will consider using ICA in preprocessing to improve the performance of our algorithm. Therefore, this research has a great impact on how to design BCI systems for neurorehabilitation in the future. Furthermore, it is also an important challenge to the actual BCI design.
